# Results from an experimental trial at a Head Start center to evaluate two meal service approaches to increase fruit and vegetable intake of preschool aged children

**DOI:** 10.1186/1479-5868-9-51

**Published:** 2012-04-30

**Authors:** Lisa J Harnack, J Michael Oakes, Simone A French, Sarah A Rydell, Farhiyah M Farah, Gretchen L Taylor

**Affiliations:** 1 Division of Epidemiology and Community Health, School of Public Health, University of Minnesota, Minneapolis, MN, USA; 2 Parents in Community Action, Hennepin County Head Start, University of Minnesota, Minneapolis, MN, USA; 3 Diabetes Unit, Center for Health Promotion, Minnesota Department of Health, Minneapolis, MN, USA; 4 Division of Epidemiology and Community Health, University of Minnesota, 1300 South 2nd St., Suite 300, Minneapolis, MN, 55454, USA

**Keywords:** Fruit and vegetable promotion, Preschool aged children, Head Start

## Abstract

**Background:**

Strategies to increase fruit and vegetable consumption of preschool aged children are needed.

**Objectives:**

Evaluate the independent effects of the following meal service strategies on intake of fruits and vegetables of preschool children: 1.) Serving fruits and vegetables in advance of other menu items as part of traditional family style meal service; and 2.) Serving meals portioned and plated by providers.

**Methods:**

Fifty-three preschool aged children completed a randomized crossover experiment conducted at a Head Start center in Minneapolis, MN. Over a six week trial period each of the experimental meal service strategies (serving fruits and vegetable first and serving meals portioned by providers) was implemented during lunch service for two one-week periods. Two one-week control periods (traditional family style meal service with all menu items served at once) were also included over the six week trial period. Childrens lunch intake was observed as a measure of food and nutrient intake during each experimental condition.

**Results:**

Fruit intake was significantly higher (p<0.01) when fruits and vegetables were served in advance of other meal items (0.40 servings/meal) compared to the traditional family style meal service control condition when they were served in tandem with other menu items (0.32 servings/meal). Intakes of some nutrients found in fruits (vitamin A and folate) were concomitantly higher. In contrast, fruit and vegetable intakes were significantly lower and energy intake significantly higher during the provider portioned compared with control condition.

**Conclusions:**

Serving fruits in advance of other meal items may be a low cost easy to implement strategy for increasing fruit intake in young children. However, serving vegetables first does not appear to increase vegetable intake. Results provide support for current recommendations for traditional family style meal service in preschool settings.

## Background

Fruit and vegetable consumption is below recommended levels for a notable proportion of preschool-aged children in the United States, with 31.5% and 80.3% consuming less than recommended amounts of fruits and vegetables respectively [1]. Consequently, strategies to encourage their consumption among this population subgroup are needed. One barrier to fruit and vegetable consumption in young children is taste preferences [2]. Humans are predisposed to prefer fatty, sweet and salty tastes, and reject sour and bitter tastes [3]. Although fruits tend to be sweet in flavor, some contain sour flavors and most are less sweet and less fatty than foods high in added fats and sugars such as chicken nuggets, candy, cakes, and sugar-sweetened beverages. Vegetables, in their minimally processed form, are not sweet nor salty flavored and some contain bitter tastes.

It is believed that children can learn to like and eat foods without highly sweet, fatty, or salty tastes through repeat exposure to them [3,4]. However, encouraging consumption of these types of foods may be difficult when they are served alongside foods that contain sweet, fatty, or salty flavors. It may be possible to address this challenge by making adjustments to the way in which foods are served.

Currently, traditional family style meal service is the recommended approach to serving meals in preschool settings [5-8]. Using this approach, all foods on the menu are served at the same time in serving bowls that are passed around the table, and children self-serve the amounts they desire. There are a number of potential advantages to traditional family-style meal service including allowing children the opportunity to self-regulate consumption (match food selection with hunger level). There are potential disadvantages as well though. Most notably, childrens tendency to prefer the taste of more energy dense foods such as tater tots and chicken fingers over less energy dense foods such as fruits and non-starchy vegetables may result in the self-selection of meals that lead to over-consumption of energy.

One potential minor adjustment to traditional family style meal service that may increase childrens intake of fruits and vegetables and decrease energy intake is to serve fruits and vegetables on the menu in advance of other meal items. Without competing foods and while hunger is at its peak, fruit and vegetable consumption could potentially be increased with this two-course traditional family style meal service approach. Furthermore, consumption of more energy-dense foods may be lower due to the satiating effect of consuming less energy dense foods (fruits and vegetables) first [9].

Another meal service approach to consider is serving meals portioned by providers rather than traditional family style. Provider portioned meals involves portioning a specific quantity of all menu items on each childs plate rather than allowing the child to self-serve food items. This approach would ensure that children consistently receive controlled quantities of fruits and vegetables along with more energy dense food items. Thus, it is possible that this approach may be more conducive to promoting fruit and vegetable intake and concomitantly moderating energy intake.

The present study was designed to evaluate the effects of these two serving strategies on fruit, vegetable, and energy intake among preschool children in a preschool setting. It was hypothesized that each of these meal service approaches (serving fruits and vegetables first and provider portioned meals) would result in higher fruit and vegetable intake and lower energy intake at the experimental meal compared with usual traditional family style meal service.

## Methods

### Overview and study design

**Figure 1:**
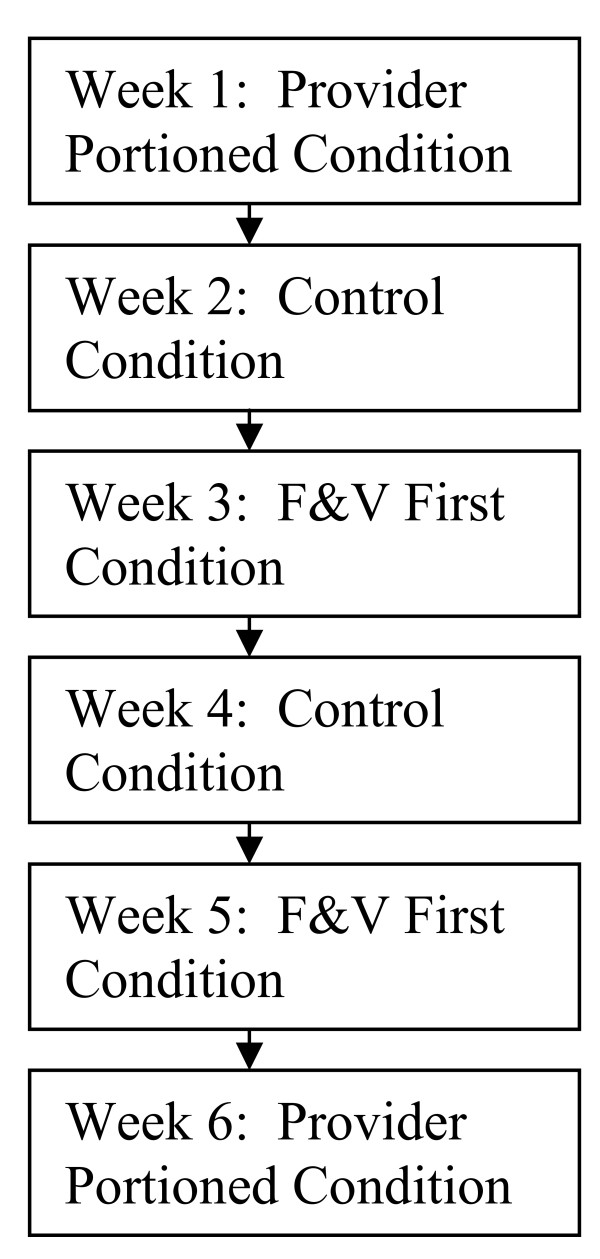
Diagram of cross-over trial design with experimental condition assignment schedule.

A single-school randomized crossover design experiment was conducted with preschool-aged children enrolled in a Head Start center in Minneapolis, Minnesota. Over a six week period, each of the two meal service approaches being evaluated (fruits and vegetables and first and provider portioned) and the control condition (traditional family style meal service) were implemented during lunch at the center. Each condition was implemented for two one-week periods over the six week period, for a total of two weeks per condition (see Figure 1). Assignment of condition by week was in random order. Childrens lunch intake was observed and recorded by trained study staff during each day of the six week period as a measure of food and energy intake during each experimental condition.

### Participant recruitment

Children in three preschool classrooms were recruited. A consent form and letter explaining the study was sent to parents. A questionnaire to collect demographic information about the parent and child was sent along with the consent form materials. A $5 gift card was provided to parents who returned the consent form, which included a check box for indicating whether or not consent was provided. A $10 gift card was provided to those who completed the questionnaire. Consent to participate was obtained for 57 of 58 children invited to participate.

Procedures followed were approved by and conducted in accordance with the ethical standards of the University of Minnesota Institutional Review Board.

### Measures

Before experimental activities were initiated, childrens height and weight were measured by study staff. Both measurements were recorded with the child in light weight clothing with shoes removed. Weight was measured to the nearest tenth of a kilogram (kg) using a digital scale. Height was measured to the nearest tenth of a centimeter using a portable stadiometer. Body mass index was calculated by dividing weight in kilograms by height in meters squared (kilograms/meter^2^). Body mass index percentiles were determined using the 2000 Centers for Disease Control and Prevention body mass index for gender and age growth chart for children 220years [10]. Using these percentiles, children were classified as overweight (8594 percentile) or obese (>95^th^ percentile).

As a measure of food and energy intake during control and experimental periods, each child was observed by study staff trained and certified in conducting lunch observations. The observation procedures were similar to those developed by Ball et al. [11]. To summarize, every day in advance of lunch, each child was given a name tag to wear that included his or her name and study assigned identification number. Observers then monitored the food consumption of children with name tags. Each observer was assigned to record consumption of up to four children. A meal observation form was used to record food consumption for each child. The form included columns for recording the types and amounts of foods taken by or served to the child. This information was recorded by the observer immediately after the child had served himself or been served. Visual estimations of food amounts were made by the observer to determine the amount taken. Any additional amounts of foods obtained by the child (e.g. seconds) or removed from the childs plate (e.g. spillage) during the meal was recorded on the form by the observer. The amount of food remaining on the childs plate after he/she was finished eating was also recorded on the form. To determine the amount remaining the observer measured leftover food using common household measuring tools such as liquid and dry measuring cups. The total amount of each food item consumed by a child was then calculated by adding together the amount taken and additional serving columns and subtracting from this total the amount of food remaining and any food recorded as being removed from the childs plate.

All observers underwent a 1.5day training to learn and practice the observation procedure. All observers passed a two-stage certification process. The first stage required that they visually estimate food portions within a specified level of accuracy. The second stage required that they demonstrate the ability to observe multiple children simultaneously and validly complete the lunch observation forms for the children observed.

As a quality control measure, throughout the experiment each completed meal observation form was reviewed by a meal observation team leader to identify mathematical errors and missing or unclear information. This review process occurred immediately following the completion of a meal observation session. The team leader corrected math errors and notified observers who made the errors. Missing or unclear information on the form was remedied where possible by asking the observer to provide the missing information or improve clarity.

The lunch observation data were entered into Nutrition Data System for Research (NDSR), a dietary analysis software program. Before the lunch observation data were entered, detailed information was collected from the food service staff at the center regarding menu items. For example, recipes were collected for foods prepared at the center and vendor product labels were obtained for pre-prepared food products. This information was used for accurate entry of the meal observation data for nutrient calculation.

### Experimental procedures

The experiment, which utilized a randomized crossover design, was conducted over a six week period within the context of the usual lunch meals served at the Head Start center. Before the experiment was initiated, a one-week run-in period was held during which study staff observed children eating during usual lunch service. The run-in period was held so that children had the opportunity to become accustomed to the presence of the lunch observers before the experiment was initiated and to mitigate the Hawthorne effect.

In the six weeks following the run-in period, each of the experimental conditions (fruits and vegetables first, provider portioned meals, and usual (control) meal service) were implemented at lunches served over two randomly assigned one-week periods (see Figure 1). The centers 13week cycle menu was running its course over the six week experimental period, hence meals items served differed from day to day during the experiment.

During each day of the control weeks, the usual traditional family style meal service approach to serving lunch meals at the center was followed. During usual lunch meals at the center children are seated around tables, and each food item on the menu is passed around the table from child to child in serving bowls for self-service. Center policy dictates that children must take some of each food item on the menu. The amount taken, however, is the childs choice. Also, the amount eaten is up to the child (leftovers are allowed). Self-served additional servings are allowed for all menu items with the stipulation that an additional serving of an item may be taken only if the first portion of that item has been completely eaten. The meal period ends when a child indicates he/she is through eating (children are dismissed one at a time from tables when they tell teacher they are through eating). Thus, there is no predetermined meal end time. At the center teachers are encouraged but not required to sit and eat with the children. Consequently, some teachers sit and eat with the children while others may do neither or one of the two.

During the fruit and vegetable first experimental weeks all fruits and non-starchy vegetables on the lunch menu were served traditional family style five minutes in advance of other menu items. Children were allowed to begin eating the fruit and vegetable items served first, with the remaining menu items (e.g. milk, entre, side dishes) placed on the tables for traditional family style meal service five minutes following distribution of the first course. All other usual meal service practices remained the same during the fruit and vegetable first experimental condition. For example, children were required to take some of each food item on the menu, the amount eaten was left up to the child (leftovers were allowed) and self-served additional servings were allowed if all the initial serving of the food was eaten. Professional judgment was used in determining that 5 minutes would be the length of time between serving fruits and vegetables and other menus items. This length of time was thought to be sufficient for the children to self-serve fruit and vegetable items on the menu and begin to eat what they had served themselves. Extending the time period to more than 5 minutes was decided against due to concerns over food safety and palatability (hot and cold items on menu would begin to lose their ideal serving temperature as they sat on a food cart awaiting service).

During the provider portioned experimental condition, a plate was prepared for each child that contained a specific quantity of each menu item. The amounts served were generally consistent with the following United States Department of Agriculture Child and Adult Care Food Program guidelines for children 35years of age: 3/4 cup milk; 1 cup juice, fruit and/or vegetable; 1/2 slice bread, 1/4 cup pasta or rice; and 1.5oz meat or meat alternative. After the plates of food were distributed, children were told to raise their hand if they had finished an item and wanted more of it. The classroom teacher served a full additional serving of items for which a request for more was made. Like during usual meal service, the amount of food eaten was up to the child (leftovers were allowed).

During each meal service condition classroom teachers held primary responsibility for providing meal-related instructions to the children, distributing/serving food, and supervising the children during the meal. During the provider portioned experimental condition a study staff member assisted the teachers in preparing the plated meals. Assistance was provided to accommodate the implementation of this more labor intensive meal service approach.

### Statistical analysis

Of the 57 children consenting to participate, four stopped attending the center or graduated to the next grade level during the study period, and were therefore excluded from all analysis. Frequencies and means were calculated for analysis to describe the demographic characteristics of study participants. Because the design is a within-person randomized experiment, measured and unmeasured confounding is mitigated. There can be no confounding (i.e., imbalance in covariates) between treatment and control conditions. Temporal influences such as social desirability bias and panel conditioning remain a threat but given the outcomes are not viewed as serious or impactful.

Because the lunch menu at the center differed from day to day across the entire experimental period, analyses were conducted to compare the nutrient and food group composition of the menus during each experimental period. To elaborate, the mean nutrient and food group composition of the lunch menus during each experimental period were calculated. Analysis of variance (ANOVA) analyses were conducted to test for possible differences between conditions.

Estimation of treatment effects (i.e., the effect of one food presentation approach over another) was performed with mixed-model regression. Generalized linear mixed models were fit for each outcome measure. Model specifications included fixed treatment effects and random effect for each person, given the within-person design.

## Results

In Table 1 the mean nutrient composition and food group servings of the centers menu during each experimental period are provided. For all nutrients and food groups examined estimates were similar between conditions (p>0.05).

**Table 1:** Comparison of mean nutrient composition and food group servings of menus during each experimental conditions

	**Control**	**F&V First**	**Provider Portioned**	**p-value**^**a**^
	**Mean (SD)**	**Mean (SD)**	**Mean (SD)**	
Energy (kcal)	426.8 (119.2)	419.6 (96.4)	468.3 (109.4)	0.56
Fat (g)	15.3 (6.2)	16.7 (6.6)	17.5 (6.0)	0.73
% Calories from Fat	31.4 (6.6)	34.5 (6.8)	32.8 (6.5)	0.58
% Calories from Saturated Fat	13.5 (3.4)	14.1 (3.7)	12.9 (2.5)	0.74
% Calories from Carbohydrate	46.3 (3.6)	44.2 (6.7)	45.4 (6.0)	0.71
% Calories from Protein	22.0 (5.1)	21.0 (2.7)	21.5 (3.5)	0.85
Total Fiber (g)	3.4 (1.2)	3.2 (1.0)	4.0 (2.0)	0.42
Vitamin A (RAE in mcg)	217.5 (58.3)	265.4 (117.3)	273.0 (143.9)	0.50
Vitamin C (mg)	17.6 (12.8)	16.7 (14.5)	16.6 (8.2)	0.98
Folate (DFE in mcg)	104.1 (42.8)	98.2 (34.6)	118.5 (39.8)	0.50
Fruit (1 cup equivalents)	0.50 (0.02)	0.44 (0.16)	0.45 (0.16)	0.56
Vegetables (1 cup equivalents)	0.86 (0.45)	0.78 (0.30)	0.77 (0.26)	0.83
Vegetables, no potatoes (1 cup equivalents)	0.76 (0.51)	0.70 (0.35)	0.65 (0.34)	0.83
Potato (1 cup equivalents)	0.11 (0.22)	0.03 (0.06)	0.16 (0.22)	0.27
Total F&V, no potatoes (1 cup equivalents)	1.26 (0.52)	1.15 (0.40)	1.10 (0.39)	0.71
Milk (1 cup equivalents)	1.01 (0.03)	1.00 (0.00)	1.01 (0.03)	0.61
Meats (1 ounce equivalents)	1.28 (0.92)	1.13 (1.13)	1.67 (0.84)	0.45
Grains (1 ounce equivalents)	1.42 (0.64)	1.32 (0.38)	1.36 (0.65)	0.92

^a^ p-values from analysis of variance.

The demographic characteristics of the study sample are provided in Table 2. The sample (n=53) was predominantly African American (75.5%). Notable proportions were obese (24.5%) or overweight (11.3%).

**Table 2:** Demographic characteristics of participants (n=53)

	**% (n)**
**Age (years)**	
2-3	50.9 (27)
4-5	49.1 (26)
**Race/ ethnicity**	
Non-Hispanic African American	75.5 (40)
Hispanic or Latina/Latino	5.7 (3)
Multi-racial	13.2 (7)
American Indian	3.8 (2)
Non-Hispanic White	1.9 (1)
**BMI for age percentile of child**	
< 85	64.2 (34)
85-94	11.3 (6)
> 95	24.5 (13)
**Education level of parent/guardian**	
Less than high school	9.4 (5)
High school graduate	41.5 (22)
Some college or associate degree	49.1 (26)

Several significant differences in food and nutrient intake were found when comparing intake during the fruit and vegetable first and control conditions (Table 3). To summarize, during the fruit and vegetable first condition intake of fruit averaged 0.40 servings per meal in comparison to 0.32 servings per meal during the control condition when fruits and vegetables were served in tandem with other menu items (p<0.01). Concomitantly, intakes of some nutrients found in fruits (vitamin A and folate) were significantly higher during the fruit and vegetable first compared to control condition. Energy intake was similar during the fruit and vegetable first and control conditions. Results were similar in analysis conducted stratified by sex and by body weight status (data not shown).

**Table 3:** Comparison of food and nutrient intake of children at lunch by experimental condition (n=53)

	**Control** **mean**	**F&V First** **mean**	**Provider** **Portioned** **mean**
Fruits (1 cup equivalents)	0.32	0.40**	0.25***
Vegetables, no potatoes (1 cup equivalents)	0.14	0.16	0.11**
Grains (1 ounce equivalents)	0.61	0.63	0.69*
Meat (1 ounce equivalents)	0.64	0.59	1.13***
Milk (1 cup equivalents)	0.45	0.41	0.51**
Energy (kcal)	223.0	237.4	284.5***
Fat (%kcal)	28.8	29.9	31.2***
Fiber (g)	2.2	2.4	2.5*
Vitamin A (RAE in mcg)	984.2	1351.4**	1212.6
Vitamin C (mg)	14.8	16.1	11.2**
Folate (DFE, mcg)	39.4	43.6*	48.9***

Note: Effects estimated from generalized linear mixed models with fixed treatment effects and a random effect for each subset.

* p<0.05.

** p<0.01.

*** p<0.001.

A number of differences in food and nutrient intake were observed when comparing intake during the provider portioned and control conditions (Table 3). Intake of grains, meat, and milk were significantly higher when meals were served provider portioned in comparison to being served traditional family style during the control condition. In contrast, intakes of both fruits and vegetables (excluding potatoes) were significantly lower when meals were served provider portioned. With respect to nutrient intake, energy intake was significantly higher when meals were served provider portioned in comparison to traditional family style (284 versus 223 kcals/meal respectively; p<0.001). Likewise, %kcal from fat, fiber, and folate were significantly higher when meals were served provider portioned. Vitamin C was the only nutrient for which intake was found to be significantly lower during the provider portioned condition in comparison to the control (traditional family style meal service) condition. Results were similar in analysis conducted stratified by sex and by body weight status (data not shown).

## Discussion

It has been contended that more healthful food consumption patterns are likely to be established if children are given the responsibility of deciding the types and amounts of foods they wish to eat from among the foods items being served [12]. Theoretically, this approach to meal service allows children to follow their internal cues to match food consumption with hunger level. Also, there is a great deal of scientific evidence suggesting that pressuring children to eat certain foods or eat all the food on their plate may be counterproductive [13-16]. It could be argued, however, that allowing self-selection of foods and food amounts in a preschool program setting will result in poor food choices by children because of their innate preference for sweet and salty foods combined with a home food environment that provides insufficient exposure to food items such as fruits and vegetables. Provider portioned meals, which involves portioning a specific quantity of all menu items on each childs plate rather than allowing the child to self-serve food items, ensures that children consistently receive controlled quantities of fruits and vegetables along with more energy dense food items. Thus, it is possible that this approach may be more conducive to promoting fruit and vegetable intake and concomitantly moderating energy intake.

Results reported in this paper are consistent with the theory that self-selection may help children match food consumption with hunger level, as energy intake was found to be lower when meals were served traditional family style in comparison to being served provider portioned. Inconsistent with the notion that provider portioning may promote consumption of foods with less preferred flavors, consumption of fruits and vegetables were lower when meals were served provider portioned. To our knowledge no previous studies have directly compared traditional family style and provider portioned meal service approaches with regard to influence on food and nutrient intake of children. Thus, findings from this study may not be corroborated.

Traditional family style meal service may have benefits beyond nutrition. Manipulating bowls and serving utensils as is required with traditional family style meal service may help preschool aged children develop fine motor skills and eye-hand perception [17-19]. Also, the act of passing food around the meal table to peers may be helpful in social development. These potential benefits are among the reasons family-style meal service is one of the feeding guidelines recommended by the National Association for the Education of Young Children [6], the Head Start Program [7], the American Dietetic Association [5], and the American Academy of Pediatrics in collaboration with the American Public Health Association [8].

Study results suggest that a two course traditional family style meal service approach in which fruits and vegetables are served in advance of other menu items may have nutritional benefits. To summarize findings, fruit consumption and intake of some nutrients found in fruits (vitamin A and folate) were found to be higher when fruits and vegetables were served first rather than in tandem with other menu items. Although the magnitude of the effect on fruit intake was modest (25% higher intake of fruit during the fruit and vegetable first in comparison to control condition), the potential cumulative effect of implementing this meal service approach across multiple meals per day and multiple days per week may be considerable. In addition, serving fruits and vegetables in advance of other menu items is a low-cost, easy to implement strategy that may be readily adapted in preschool settings.

Vegetable intake was not found to be significantly higher during the fruit and vegetable first in comparison to control condition. This finding may be due to the fact that vegetables, in minimally processed form, generally do not contain favored flavors (they are neither sweet nor salty flavored) and some contain bitter tastes. As a result, increasing their consumption may require changes beyond manipulating the order in which they are served. Preparation method is an additional change to consider, as the acceptability of vegetables to children may be improved by avenues such as serving fresh vegetables with dip, preparing cooked vegetables with some added fat, and incorporating vegetables covertly in entrees [20-24]. In our study the vegetables served were predominately canned vegetables that may have lacked taste and texture appeal. In addition, the fresh vegetables on the menu were not consistently served with a dip. It could be speculated that serving vegetables in advance of other menu items may be an effective strategy for increasing vegetable consumption if attention is given to maximizing taste appeal.

Strengths of the proposed study are numerous. The study was conducted in a Head Start center so that results are relevant in the context of a preschool program that reaches children who are at high risk for overweight and obesity. The experiment was conducted in a naturalistic setting using the cycle menu in place at the participating center. Thus, external validity of findings is likely high. As a trade-off, there may be concerns with internal validity as potential confounding factors such as pre-meal hunger level were not controlled. The use of randomization in tandem with repeat exposure to each experimental condition (2 one week periods for a total of 10 experimental meals per condition) reduces concern over potential confounders because these factors are apt to be equally distributed across experimental periods. Additional weaknesses include potentially limited generalizability of findings because the study was conducted in one Head Start center located in an urban area. Given the small-scale nature of the study, statistical power to examine possible differences in response by factors such as bodyweight status, race, and sex was limited. A final methodological limitation that should be noted is the reliance on meal observation data as the primary measure of food intake. With the meal observation methodology food amounts may be misestimated by observers, thereby introducing measurement error. Assuming misestimating occurred similarly for each experimental condition, the most likely outcome of this measurement error would be failure to detect effects (type II error).

## Conclusions

Results suggest that serving fruits in advance of other meal items may be a low-cost and easy to implement strategy to increase fruit intake of preschool aged children. Also, results provide support for use of traditional family style meal service in preschool centers in communities where overweight and obesity are childhood nutrition concerns. Additional studies in larger and more diverse samples and in other settings such as the home are needed to verify findings.

## Competing interests

Author disclosure: L.J. Harnack, J.M. Oakes, S.A. French, S.A. Rydell, F.M. Farah, G.L. Taylor, no competing interests.

## Authors contributions

All of the authors were involved in designing the research. L.J.H. and S.A.R. conducted the research. S.A.R. and J.M.O. had primary responsibility for analyzing the data. All of the authors were involved in interpreting results from the analyses. L.J.H had primary responsibility for writing the paper, with all of the authors contributing by reviewing and editing drafts of the manuscript. All authors read and approved the final manuscript.
